# The emergence of *Leptospira borgpetersenii* serovar Arborea in Queensland, Australia, 2001 to 2013

**DOI:** 10.1186/s12879-015-0982-0

**Published:** 2015-06-14

**Authors:** Colleen L. Lau, Chris Skelly, Michael Dohnt, Lee D. Smythe

**Affiliations:** Queensland Children’s Medical Research Institute, Brisbane, Australia; WHO Collaborating Centre for Children’s Health and Environment, The University of Queensland, Brisbane, Australia; Research School of Population Health, Australian National University, Canberra, Australia; Freelance Consultant, Brisbane, Australia; WHO/FAO/OIE Collaborating Centre for Reference and Research on Leptospirosis, Forensic and Scientific Services, Health Support Queensland, Department of Health, Brisbane, Australia

**Keywords:** Leptospirosis, Leptospira, Emerging infectious diseases, Infectious disease epidemiology, Infectious disease outbreaks, Zoonoses, Environmental health, Eco-epidemiology, Tropical medicine

## Abstract

**Background:**

Leptospirosis is an emerging infectious disease, with increasing frequency and severity of outbreaks, changing epidemiology of populations at risk, and the emergence of new serovars. Environmental drivers of disease transmission include flooding, urbanisation, poor sanitation, changes in land use and agricultural practices, and socioeconomic factors. In Queensland, human infection with *Leptosira borgpetersenii* serovar Arborea was first reported in 2001. This study aims to report the emergence of serovar Arborea in Queensland from 2001 to 2013, and investigate potential risk factors for infection and drivers of emergence.

**Methods:**

Data on laboratory-confirmed cases of human leptospirosis in Queensland were obtained from the enhanced surveillance system at the WHO/FAO/OIE Collaborating Centre for Reference and Research on Leptospirosis in Brisbane, Australia. The changing epidemiology of serovar Arborea from 2001 to 2003 was described with respect to case numbers, proportion of leptospirosis cases attributed to the serovar, and geographic distribution. Differences in risk factors for the most common serovars were compared.

**Results:**

During this period, 1289 cases of leptospirosis were reported, including 233 cases attributed to serovar Arborea. Risk factors for infection include male gender (91 % of cases), occupation, and recreational exposure. Most common occupations recorded were banana workers (28.4 %), meat workers (7.2 %), dairy farmers (5.8 %), graziers/stockmen (5.5 %), ‘other agricultural/rural workers’ (16.4 %), and tourists or tourism operators (4.6 %). Time trend analysis showed that while non-Arborea cases decreased over the study period, Arborea cases increased by 3.4 cases per year. The proportion of annual cases attributed to Arborea peaked at 49 % in 2011 after unprecedented flooding in Queensland. Mapping of cases by residential location showed expansion of the geographic range of serovar Arborea, concentrating mostly around Brisbane, Cairns and Innisfail. Serovars varied significantly between ages and occupational groups, and serovar Arborea was most strongly associated with ‘other agricultural/rural workers’.

**Conclusions:**

*Leptospira borgpetersenii* serovar Arborea has been emerging in Queensland since 2001, with increase in case numbers, the proportion of leptospirosis infections attributed to the serovar, as well as expansion of its geographic distribution. Reasons for this emergence are unknown, but climatic factors and environmental change are likely to have played important roles.

**Electronic supplementary material:**

The online version of this article (doi:10.1186/s12879-015-0982-0) contains supplementary material, which is available to authorized users.

## Background

Leptospirosis is caused by infection with bacteria that belong to the phylum Spirochaetes and genus *Leptospira*, with over 250 known pathogenic serovars [1]. Leptospirosis is an emerging endemic and epidemic disease of global importance, with recent reports of increasing frequency and severity of outbreaks, changing epidemiology of populations at risk, and the emergence of new serovars [2–4]. Environmental drivers of disease transmission and infection vary between different epidemiological settings (e.g. agricultural areas, urban slums, tropical islands), and include climate, land use, international trade, animal reservoirs, agricultural practices, as well as socioeconomic and cultural factors [2–5]. It has become increasing apparent that rainfall, extreme weather, flooding, urbanisation, and recreation are important factors for leptospirosis emergence [2, 3, 6–8], that biodiversity might be protective [9], and ecological changes could influence serovar emergence [10].

The state of Queensland in Australia has one of the highest incidences of leptospirosis in the developed world [7], with the majority of cases reported in the wet summer months [11]. Within Queensland, there is significant geographic variation in incidence, with the highest risk in the tropical northern parts of the state. From 2001 to 2007, Innisfail (a banana and sugarcane growing area in the far north of Queensland) recorded an average of 119 cases/100,000/year (range 81 to 157/100,000/year), compared to an average of 7.9/100,000/year in the regional city of Cairns, and <0.5/100,000/year in metropolitan Brisbane, the state’s capital city in the southeast [12]. Approximately 60 % of cases required hospitalisation, but deaths were only reported very occasionally. In Australia, there has also been a recent shift in disease epidemiology with increasing number of cases acquired through recreational exposure and/or international travel [7].

*Leptosira borgpetersenii* serovar Arborea was originally described in Italy in 1955 [13]. Since then, infection has been reported in humans and animals in diverse environmental, ecological, and cultural settings including Barbados [14], French West Indies [15], Argentina [16], Azores [17], Slovakia [18], New Zealand [19], and Australia [11, 20, 21]. Rodents, particularly *Mus domesticus* and *Rattus rattus,* are the predominant animal reservoirs for serovar Arborea.

In Australia, the first human infection with serovar Arborea was identified in 1998 in northern New South Wales (the state immediately south of Queensland), and cases in Queensland have been reported since 2001 [11, 20]. Infection with serovar Arborea has also been identified in mammal species across Australia, including rodents in Queensland [11, 20], brushtail possums in urban Sydney [21], and dogs in animal shelters across Australia [22].

In this paper, we describe the changing epidemiology of human leptospirosis infections in Queensland from 2001 to 2013, with particular focus on the increasing incidence and geographic distribution of serovar Arborea, and discuss possible drivers and future implications of its emergence.

## Methods

### Data sources

#### Queensland cases

Data on laboratory-confirmed cases of human leptospirosis in Queensland from 2001 to 2013 were obtained from the enhanced surveillance system at the WHO/FAO/OIE Collaborating Centre for Reference and Research on Leptospirosis in Brisbane, Australia. Where possible, data were collected on demographics, place of residence by postcode, occupation, and overseas travel. All laboratory-confirmed cases from January 2001 to December 2013 who resided in Queensland postcode areas were included in this study. Occupational groups were divided into seven categories: meat workers, dairy farmers, banana workers, grazier/stockmen, other agricultural/rural workers, tourists/tourist operators, and others/unknown. Unfortunately, data on overseas travel were only obtained from 32.4 % of cases and destination history was insufficient for meaningful analyses.

#### Laboratory confirmation

Leptospirosis is a notfiable disease in Queensland, and at our reference laboratory, one or more of the following criteria are taken to indicate confirmed acute infections [23]:

i. Isolation of leptospires from blood cultures
ii. Fourfold rise in antibody titres using the microscopic agglutination test (MAT)
iii. Single titre of ≥ 1:400 using the MAT

The diagnostic test(s) used varied between cases, and depended on requests by treating clinicians and number of days since the onset of illness. From 2008, molecular diagnosis (PCR) was also used at the laboratory, but those cases not confirmed by isolation and/or MAT were not included in this study because these were considered to be probable rather than confirmed cases. For some cases, follow-up serum samples were not available and any rise in antibody titres could not be determined. For such cases, a single MAT titre of ≥ 1:400 was used to indicate acute infection.

#### Determination of serovars associated with confirmed cases

Wherever available, isolations were used to determine serovars responsible for infections. For cases where isolations were not available (including cases where culture was not requested by the treating clinician, or cultured not performed because cases presented after the bacteraemic phase of the illness, or culture did not yield any isolations), the putative serovar was determined by MAT using the routine panel of 22 serovars listed in Additional file 1. The MAT is currently the reference serological test recommended by the WHO [24] and the International Committee on Systematic Bacteriology, Subcommittee on the Taxonomy of *Leptospira*, and is considered to be serogroup rather than serovar specific [25]. However, in areas where there are comprehensive data available on the range of serovars that are circulating locally (such as in Queensland), the MAT could be used to determine the putative serovar with much greater specificity. Limitations of the MAT include cross-reactions between serovars within a serogroup, and complex paradoxical reactions in persons who have had previous infections [25]. Whilst noting such limitations, the MAT is the recognised gold standard method for determining putative serovars where cultures were not performed or leptospires not isolated [24].

Prior to 2001, serovar Ballum was included in our laboratory’s routine MAT panel to represent the Ballum serogroup. Serogroups are no longer used in the taxonomic classification of *Leptospira*, but they remain useful for laboratory and epidemiological purposes [1]. Although serovars from the Ballum serogroup had never previously been isolated or identified on MAT in cases that were locally acquired in Queensland, the serovar was included in our routine MAT panel to allow identification of cases acquired overseas and/or local emergence of new serovars. In 2001, *Leptosira borgpetersenii* serovar Arborea was isolated from a human – the first ever isolation of a serovar in the Ballum serogroup for leptospirosis cases that were acquired locally in Queensland [11, 20]. From 2001, serovar Arborea replaced serovar Ballum as the representative of the Ballum serogroup in the routine MAT panel used in our laboratory. Since then, serovar Arborea has been isolated from both human and animal samples [11, 20, 21], and confirmed using the cross-agglutination absorption test (CAAT) [24, 26]. Considering that serovar Arborea was the only member of the Ballum serogroup that has ever been isolated from Queensland, positive MAT reactions to serovar Arborea were presumed to be associated with this serovar.

For this study, cases from 2001 to 2004 were only attributed to serovar Arborea if they were confirmed by isolation or specifically noted to be serovar Arborea in the database. However, it was likely that some of the cases attributed to serovar Ballum during the early years of the study period were actually caused by serovar Arborea. Even though there were only small numbers of cases attributed to serovar Ballum, we have reported them separately to highlight possible additional cases of Arborea that were not included in our statistical analyses.

#### Climate, population, and employment statistics

Data on monthly rainfall for Brisbane and Cairns were obtained from the Australian Government Bureau of Meteorology [27]. Australian population and employment data were obtained from the Australian Bureau of Statistics [28].

#### Maps

Queensland is the second largest state in Australia, with a total land area of over 1.73 million km^2^, and Brisbane the third largest city in Australia. Large population centres in Queensland are mostly situated in subtropical and tropical coastal areas (including the capital city of Brisbane with a population of ~2 million), while most inland areas are arid and very sparsely populated. Queensland’s state and postcode boundaries were obtained from the Australian Bureau of Statistics. Postcode boundaries are largely informally defined areas, and modified as the demands of postal delivery change over time. Consequently, postcode-georeferenced cases from 2001 were linked to 1996 postcode boundaries, cases from 2002 to 2006 linked to 2001 postcode boundaries, cases from 2007 to 2011 linked to 2006 boundaries, and cases from 2012 to 2013 linked to 2011 boundaries.

**Fig. 1:**
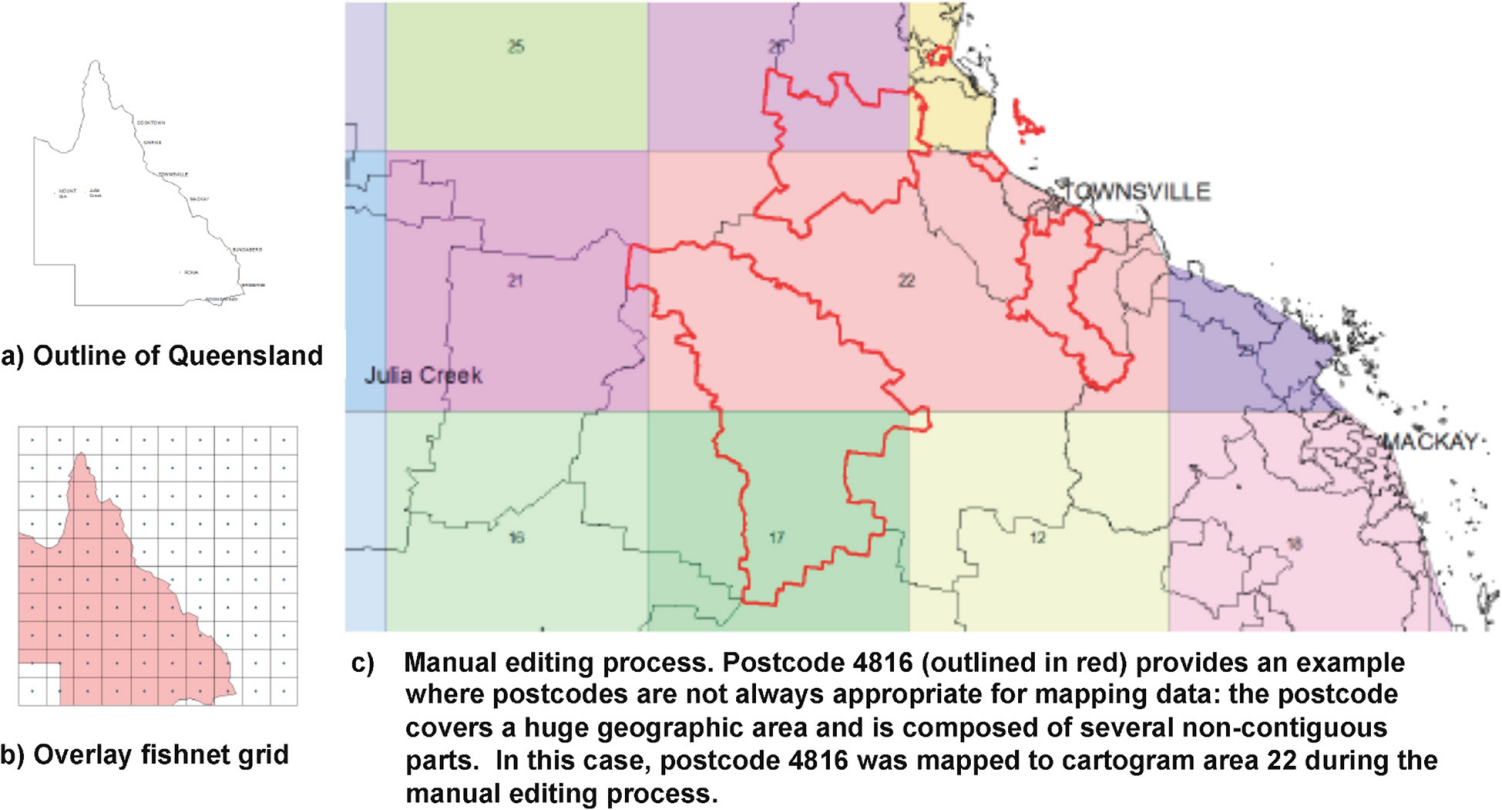
Methods used to design a multi-region cartogram used to summarise postcode areas. **a** creating a simple outline of Queensland, **b** creating and overlaying a fishnet grid, and **c** manually editing postcode areas that overlapped multiple cartogram regions

There are over 450 postcodes in Queensland that vary significantly in geographic area coverage, and mapping case data by postcodes or postcode centroids could therefore produce maps that distort the true location and density of cases. For example, Fig. 1c shows a postcode that covers a very large area and is composed of multiple non-contiguous parts. In order to improve the representation of the geographic location and number of cases across the state, we designed a cartogram to summarise postcode areas into approximately 30 regions. An outline of Queensland (Fig. 1a) was overlaid with a 'fishnet' grid to provide the underlying pattern of cartogram regions (Fig. 1b). Over 90 % of postcode areas fell within one cartogram region. For the small number of postcodes areas that overlapped multiple cartogram regions, a manual editing process was used to determine the region that most accurately represents that postcode (see example in Fig. 1c). Some cartogram regions were combined because they were overlapped by very large postcode areas.

Maps of reported cases were produced separately for Arborea and non-Arborea serovars:

i. Overview maps to show the geographic distribution of all leptospirosis cases in Queensland during the study period (2001 to 2013) by cartogram region;
ii. Maps of the crude incidence rate per cartogram region, calculated using the number of cases reported in each region and the estimated population from the 2011 national census; and
iii. Time-series maps to demonstrate changes in case numbers and location over the study period, calculated using reported cases per cartogram for each calendar year.

Spatial data were collated, stored, linked and mapped using the geographic information systems (GIS) software, ArcMap v10.0 (Environmental Systems Research Institute, Redlands, California).

### Statistical analysis

Chi-squared tests or Fisher exact tests were used to compare differences between categorical groups. For comparisons between cases attributed to Arborea and ‘non-Arborea’ serovars, the ‘non-Arborea’ group included cases attributed to serovar Ballum, other serovars, and undertermined serovars. For occupational groups, univariate logistic regression analysis was performed and odds ratios (OR) calculated for occupational groups with infections attributed to each of the most common serovars. We examined the time trend of reported cases over the study period using a least squares regression of annual counts, and produced an estimate of the linear change in case numbers over the study period (regression line slope), which we qualified with 95 % confidence intervals (CI). STATA v11.1 software (StataCorp, College Station, Texas) was used for statistical analyses, and *p* values of <0.05 were taken to indicate statistical significance.

### Ethics approval

Permission to use de-identified data for this research was obtained from the Human Research Ethics Committee at Queensland Health Forensic and Scientific Services (Application 290711).

## Results

**Fig. 2:**
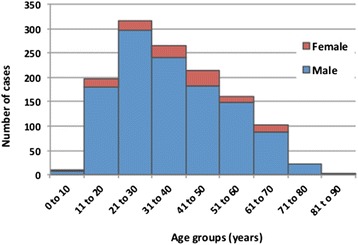
Age and sex distribution of leptospirosis cases reported in Queensland from 2001 to 2013

During the 13 year study period from January 2001 to December 2013, 1289 confirmed cases of acute leptospirosis were diagnosed in Queensland, equivalent to an average of 99.2 cases per year. Cases were predominantly diagnosed in males (*n* = 1172, 91 %), with only 117 (9 %) of cases reported in females (Fig. 2). Cases were reported in all ages, with highest numbers in the 21 to 30 year old age group (*n* = 316, 25 %), and 74 % of cases were reported in the working age groups from 21 to 60 years (Fig. 2).

A total of 233 confirmed cases were attributed to *L. borgpetersenii* serovar Arborea and 32 cases to *L.borgpetersenii* serovar Ballum, accounting for 18.1 % and 2.5 % of confirmed cases respectively. Other serovars accounted for 973 cases (75.5 %), and the most commonly reported were *L. interrogans* serovar Zanoni (271 cases, 21.5 %), *L. interrogans* serovar Australis (194 cases, 15.4 %), and *L. interrogans* serovar Hardjo (148 cases, 11.7 %). Serovar was unknown or unable to be determined in 51 cases (4.0 %).

During the study period, 452 individual serovar isolations were recorded from locally-acquired infections, representing 35.1 % of confirmed cases. Of the 233 cases attributed to serovar Arborea, 63 (27.0 %) were confirmed by isolation, and the remainder were putatively assumed to be associated with serovar Arborea based on MAT results. Appendix B lists the 14 serovars that have been isolated from humans who acquired leptospirosis in Queensland from 2001 to 2013. Notably, serovar Arborea is the only serovar in the Ballum serogroup that has ever been isolated in cases acquired in Queensland.

**Fig. 3:**
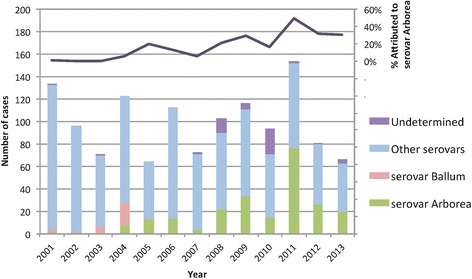
Annual reported cases of leptospirosis attributed to serovar Arborea, serovar Ballum, other serovars, and undertermined serovars; and proportion of total infections attributed to serovar Arborea, Queensland 2001 to 2013

**Fig. 4:**
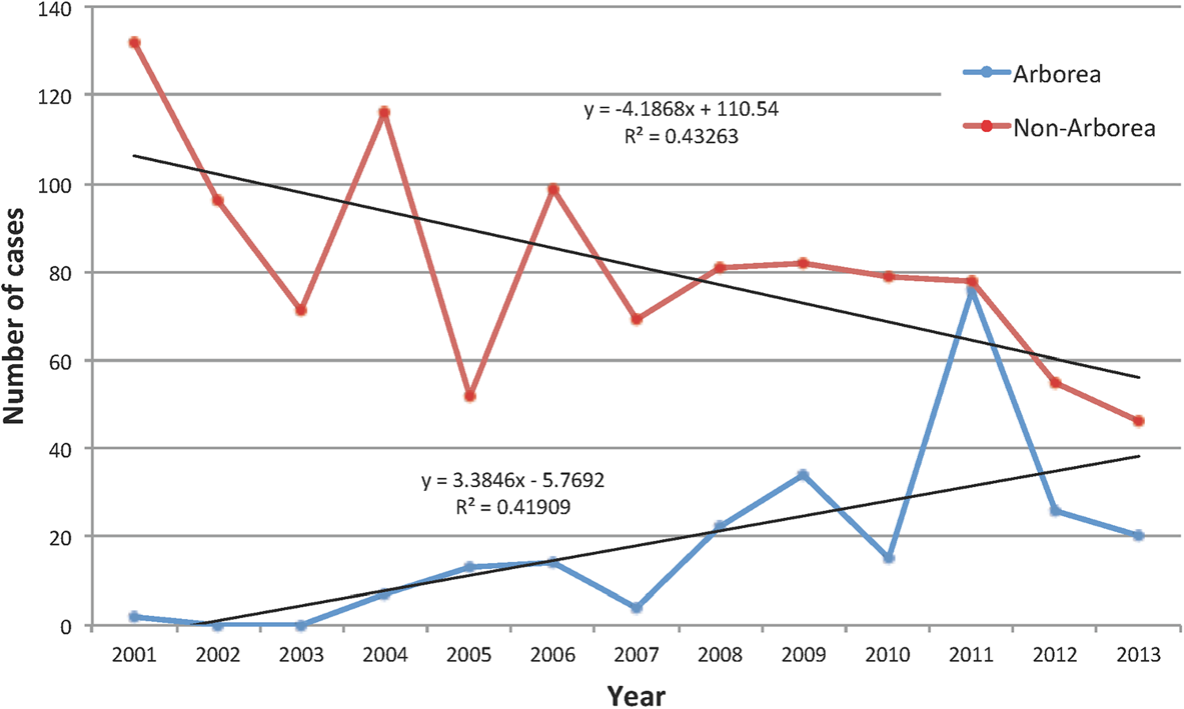
Temporal trend (with linear trend lines) of leptospirosis cases attributed to serovar Arborea versus non-Arborea in Queensland, 2001–2003

The number and proportion of annual cases attributed to serovar Arborea increased over the study period, peaking at 49 % of total cases in 2011 (Fig. 3). Annual numbers attributed to serovar Ballum, other serovars, and undetermined cases are also shown on Fig. 3. While the annual number of non-Arborea cases declined over the study period, Arborea case numbers significantly increased (Fig. 4). The linear time trend over the study period shows that serovar Arborea (R^2^ = 0.42) increased at a rate of 3.38 cases per year (95 % CI 2.75–3.72), while non-Arborea serovars (R^2^ = 0.43) taken as a single group decreased at a rate of 4.18 cases per year (95 % CI 4.27–5.07) (Fig. 4). There was significant inter-annual variation in the data affecting the explanatory value of the trend (R^2^ value). For example, if the 2011 datum point (major flooding across Queensland) was removed, the R^2^ for Arborea cases would increase to 0.63.

**Fig. 5:**
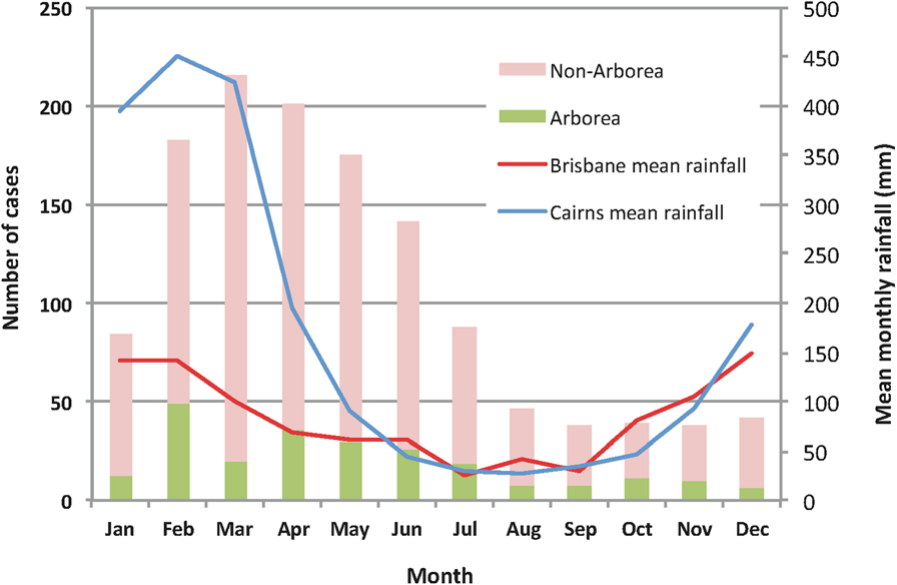
Month of diagnosis of leptospirosis cases (Arborea and non-Arborea serovars) reported in Queensland from 2001 to 2013, and mean monthly rainfall (mm) in Brisbane (southeast Queensland, population ~2 million) and Cairns (northeast Queensland, population ~150,000)

Overall, 81.5 % of 233 serovar Arborea cases and 84.9 % of 1056 non-Arborea cases were reported from January to July (Fig. 5), which coincides with the months during and following Queensland’s wet summer season, when storms, cyclones, and flooding occur most frequently, particularly in the far northern tropical parts of Queensland.

**Fig. 6:**
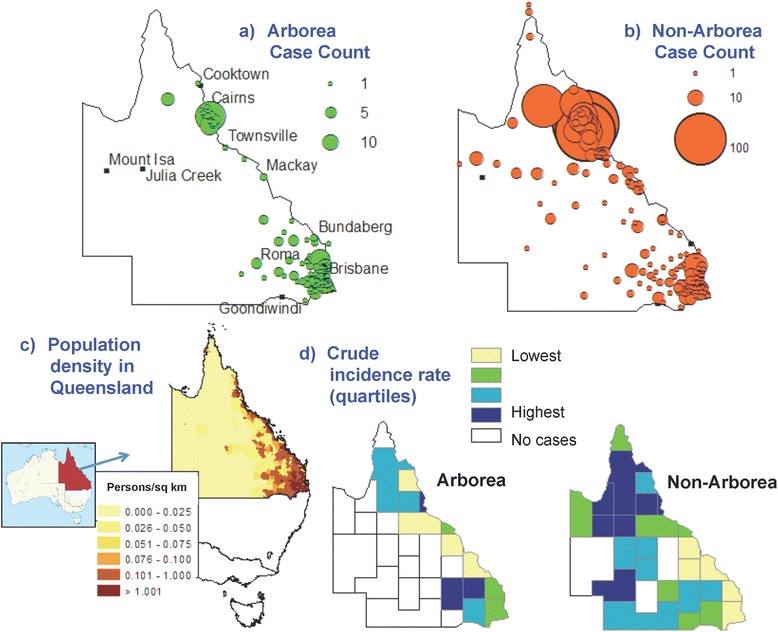
Geographic distribution of total reported leptospirosis cases in Queensland from 2001 to 2013. **a & b** Cases attributed to Arborea and non-Arborea serovars respectively by cartogram regions, **c** Population distribution in the state of Queensland, Australia, **d** Cartogram regions with crude incidence rates for Arborea and non-Arborea serovars (presented in quartiles)

**Fig. 7:**
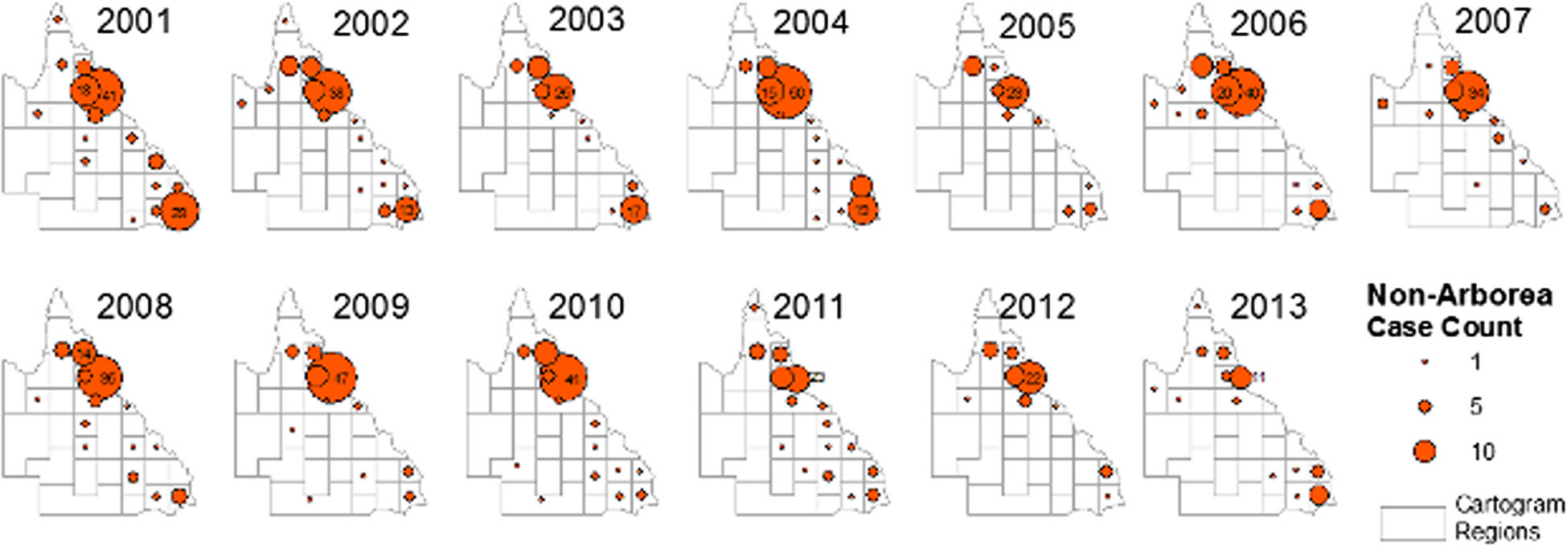
Annual reported cases attributed to non-Arborea serovars in Queensland, 2001 to 2013. Proportional symbols represent the number of cases reported per cartogram region

**Fig. 8:**
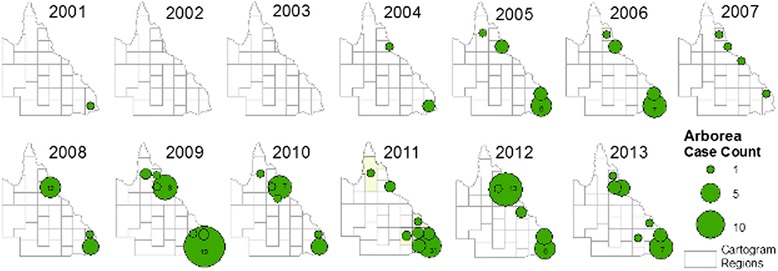
Annual reported cases attributed to serovar Arborea in Queensland, 2001 to 2013. Proportional symbols represent the number of cases reported per cartogram region

The maps in Fig. 6a and b show the geographic distribution of all reported cases from 2001 to 2013, stratified by Arborea and non-Arborea serovars. To put the geographic distribution of cases into perspective and aid interpretation of findings, Fig. 6c shows the location of Queensland in Australia, and the population distribution across the state. Agricultural areas are generally located in coastal regions in areas with medium to high population density. In cartogram regions with reported cases, crude incidence rates were calculated for Arborea and non-Arborea infections, and shown as quartiles in Fig. 6d. The annual reported cases attributed to non-Arborea and Arborea serovars are shown in Figs. 7 and 8 respectively, with proportional symbols representing the number of cases per cartogram region.

Occupation was recorded in 712 cases (55.2 %), and of these, 202 (28.4 %) were banana workers, 51 (7.2 %) meat workers, 41 (5.8 %) dairy farmers, 39 (5.5 %) graziers/stockmen, and 117 (16.4 %) other agricultural/rural workers (Table 1). To put the above findings into perspective, the male to female ratio of agricultural workers in Australia in 2012 was 2.05, and the proportion of the total Australian workforce employed in the agricultural industry was 3.5 % for males and 2.0 % for females [28]. Thirty-three cases (4.6 %) were diagnosed in tourists or those who worked in the tourism industry, including seven who listed ‘white water rafting’ as their main occupation. Fourteen cases (2.0 %) were reported from military personnel.

**Table 1:** Univariable logistic regression of occupational groups associated with infection attributed to different leptospiral serovars, Queensland 2001 to 2013

Serovar			Arborea	Australis	Zanoni	Hardjo
Number of cases attributed to serovar			233	194	270	147
Occupation	N	% of Total	Odds Ratio^a^ (95 % CI)	Odds Ratio^a^ (95 % CI)	Odds Ratio^a^ (95 % CI)	Odds Ratio^a^ (95 % CI)
Others/unknown	806	62.5 %	1	1	1	1
Meat workers	51	4.0 %	**0.08 (0.01–0.58)**	no cases	**0.18 (0.04–0.74)**	**7.62 (4.16–13.94)**
Dairy farmers	41	3.2 %	**0.21 (0.05–0.86)**	no cases	**2.01 (1.02–3.98)**	**5.21 (2.61–10.37)**
Banana workers	202	15.7 %	**0.39 (0.23–0.66)**	**1.62 (1.11–2.36)**	**2.96 (2.12–4.13)**	**0.31 (0.13–0.72)**
Grazier/Stockmen	39	3.0 %	0.88 (0.38–2.02)	0.14 (0.02–1.01)	0.36 (0.11–1.19)	**7.76 (3.94–15.27)**
Other agricultural/rural workers	117	9.1 %	**1.78 (1.16–2.73)**	0.54 (0.28–1.03)	**0.41 (0.21–0.79)**	1.48 (0.82–2.67)
Tourists/tourism operators	33	2.6 %	1.28 (0.57–2.90)	0.72 (0.51–2.07)	1.63 (0.74–3.57)	no cases
Total	1289	100.0 %				

^a^Statistically significant odds ratios shown in bold

**Fig. 9:**
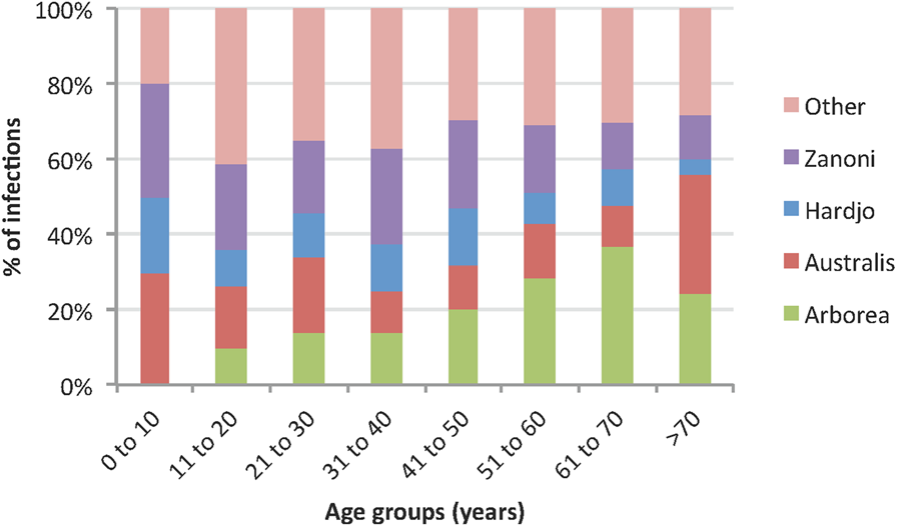
Variation in serovar distribution of reported cases between age groups, Queensland 2001 to 2013

Serovars associated with infections varied significantly between occupational groups. Serovar Arborea was significantly more common in other agricultural/rural workers; serovar Australis in banana workers; serovar Zanoni in banana workers and dairy farmers; and serovar Hardjo in meat workers, dairy farmers, and graziers/stockmen. Table 1 shows the results of univariate logistic regression analyses of serovars associated with occupational groups, and the odds ratios (with 95 % confidence intervals) for each group to be diagnosed with an infection associated with each of the most common serovars. Serovar distribution also varied significantly between age groups (Pearson chi-square = 84.3, *p* = 0.000), as shown in Fig. 9, and the proportion of cases attributed to serovar Arborea differed between ages (Pearson chi-square = 54.3, *p* = 0.000).

## Discussion

Human leptospirosis in Queensland is associated with strong seasonal variation in incidence (Fig. 5), and risk factors for infection include male gender (Fig. 2), occupational exposure, and recreational exposure (Table 1). These results corroborate findings from many epidemiological studies of leptospirosis around the world. Variation in serovar distribution between age groups (Fig. 9) and occupations (Table 1) suggest that multiple risk factors and exposure pathways are at play, and differ between demographic groups.

Slack et al. reported the emergence of serovar Arborea in Queensland up to 2004 [11], and our study shows that Arborea has continued to emerge since that time, with increase in total case numbers, proportion of total infections attributed to this serovar (Figs. 3 and 4), as well as expansion of the serovar’s geographic distribution (Fig. 8).

Geospatial mapping showed that geographic distribution of both Arborea and non-Arborea cases varied across Queensland. Figure 6a shows that cases associated with serovar Arborea infection were mostly concentrated in the southeast coastal corner near Brisbane, and the area around Cairns and Innisfail in the northeast, and very few cases were reported from inland regions. Figure 6b shows that cases associated with non-Arborea serovars were predominantly diagnosed in the Cairns and Innisfail area, but cases were also found in the southeast coastal areas as well as more arid inland areas of the state. Figure 6c shows that locations of high crude incidence rates differed for Arborea and non-Arborea cases. Although there were insufficient cases to produce meaningful maps of annual incidence rates by cartogram regions, visual inspection shows no dramatic changes in the incidence or geographic distribution of non-Arborea serovars over the study period (Fig. 7). On the other hand, Fig. 8 shows that since its initial detection in southeast Queensland in 2001, serovar Arborea has gradually increased in both case numbers and geographic range.

The reasons for the recent emergence of serovar Arborea are currently unknown, but differences in risk between age groups, occupations, geographic location, and annual variation in incidence of Arborea versus non-Arborea serovars suggest that both behavioural and environmental factors play important roles in disease transmission. Possible environmental drivers of the emergence of serovar Arborea in Queensland over the past decade include population growth, agricultural intensification and associated deforestation, alterations in land use, and changing climatic patterns and extreme weather events. Both the Brisbane and Cairns/Innisfail areas are relatively densely populated compared to other parts of Queensland, and are surrounded by areas of intense agriculture and farming. Urbanisation is also invariably associated with the proliferation of rodents, which have been found to be carriers of serovar Arborea in Australia [20]. Globally, environmental degradation, biodiversity loss, and ecosystem stress have been linked to the emergence of infectious diseases [29], and these factors could all potentially have played a role in the recent emergence of serovar Arborea in Queensland. Considering that there are molecular similarities between Australian and European isolates of serovar Arborea, and that it had never been isolated in Australia prior to 1998, the serovar may have been introduced in recent years from international cargo ships [20], but there is no evidence to support this hypothesis.

The strong seasonal variation in incidence (Fig. 5) suggests that climate (including rainfall, flooding, and temperature) is an important driver of leptospirosis transmission in Queensland. In 2010/2011, Australia experienced one of the strongest La Nina events and wettest summers on record. From September 2010 to March 2011, the country received double its average rainfall, which resulted in widespread severe flooding and destruction of both urban and natural environments [30]. In addition, Category 5 Cyclone Yasi made landfall near Cairns in northern Queensland in February 2011, further exacerbating the already severe flooding and destruction. Natural disaster was declared in over 99 % of Queensland (total land area of 1.73 million km^2^) and the capital city of Brisbane experienced its worst flooding since 1974 [30]. The 2011 summer of natural disasters in Queensland was followed by an unprecedented number of leptospirosis infections, and the highest recorded proportion of infections (49 %) attributed to serovar Arborea (Fig. 3) [31, 32].

Globally, many leptospirosis outbreaks have been reported after flooding [2], but few have specifically examined the emergence of serovars associated with floods. Possible links between severe flooding and the emergence of a serovar include the proliferation of specific species of rodents that serve as reservoir hosts of the serovar; changes in environmental conditions (e.g. temperature, humidity, pH) that favour the survival of the serovar in water and soil; and increased contact between humans, animals, and floodwaters during the aftermath of disasters.

Our findings should be interpreted in light of the limitations of this study. Surveillance data recorded postcodes at each individual’s place of residence, but infections could have occurred at work, during travel to other parts of the state or country, or even overseas. Data on international travel were incomplete, and it is possible that some of the cases were acquired overseas. In Fig. 5, rainfall data were only shown for Brisbane and Cairns, but as seen in Fig. 6, many cases were reported from areas of Queensland that are far from either of these cities. Occupation was only recorded in 55.4 % of cases and it is possible that some of cases with ‘unknown occupation’ belonged to the occupational categories used for statistical analysis, thus weakening the strength of association (odds ratios and confidence intervals) reported. Results on demographics, occupation, and annual/monthly case numbers were aggregated for the whole state of Queensland, and more stratified data analysis might produce further insights into risk factors and disease ecology. Although census population data from 2011 were used to calculate crude incidence rates in each cartogram region over the entire study period (Fig. 6d), significant population growth has occurred over the study period. Cartogram regions do not correspond to administrative zones (Fig. 1), and accurate calculation of incidence was not possible, particularly with the small number of cases reported in some regions. Crude incidence rates were therefore only reported as quartiles rather than quantitative estimates (Fig. 6d).

Limitations of the MAT have already been noted, and as mentioned earlier, some of cases recorded as serovar Ballum could have been caused by serovar Arborea, but the small numbers involved (shown in Fig. 3) would not alter the overall findings or implications of our study. During the study period, the successful isolation of serovar Arborea from 63 cases (27 %) provides substantial evidence that the serovar is endemic in Queensland. Although 73 % of confirmed cases attributed to serovar Arborea were based on MAT alone, the absence of other serovars in the Ballum serogroup in Queensland (Additional file 2) significantly improves the MAT’s specificity for serovar Arborea. The database used in the earlier years of the study period recorded the number of confirmed cases attributed to each serovar, but did not allow accurately determination of the exact number of cases confirmed by each diagnostic test. The diagnostic test(s) performed varied between cases (depending on requests by clinicians and the time since onset of illness), some cases were confirmed by more than one diagnostic test, and isolations were not attempted in all cases. However, the proportion of cases confirmed by each diagnostic test does not change the overall findings or implications of our study.

If the environmental factors discussed above were indeed important in the emergence of serovar Arborea in Queensland, leptospirosis incidence could potentially escalate in the future with population growth, agricultural intensification, and increase in extreme weather events associated with climate change. Ongoing disease surveillance is therefore important for monitoring the evolution in incidence and geographic distribution of serovar Arborea, as well as early detection of possible emergence of other serovars. In high-risk areas, and particularly during the high-risk season, awareness of leptospirosis should be raised with the public, agricultural and tourism industries, and clinicians.

The transmission dynamics of leptospirosis are highly complex, and future research should aim to adopt an eco-epidemiological approach to explore the interactions between humans, animals, and the environment in determining overall infection risk and serovar emergence. Zoonotic diseases are responsible for the majority of emerging infectious diseases [29], and improved understanding of disease ecology would provide an evidence base to guide the development of tools to help predict the timing and triggers for outbreaks, determine hotspots based on environmental factors, identify subpopulations who are at greatest risk. Such tools will in turn help inform mitigation strategies, early warning systems, and public health interventions to reduce leptospirosis disease burden.

## Conclusions

*Leptospira borgpetersenii* serovar Arborea has been emerging in Queensland since 2001, with increase in case numbers, the proportion of leptospirosis infections attributed to the serovar, as well as expansion of its geographic distribution. Reasons for this emergence are currently unknown, but climatic factors (especially flooding) and environmental change are likely to have played important roles.

## Availability of supporting data

We are unable to make the raw data freely available because some Queensland postcode areas are very sparsely populated, and cases could potentially be personally identified by age, sex, occupation, and date of diagnosis of leptospirosis. Requests for raw data should be made to the Research and Human Ethics Coordinator (+61 7 3000 9363), Forensic and Scientific Services, Health Support Queensland, Department of Health, Queensland Government.

## Additional files

Additional file 1:
**Leptospiral serovars used in the routine microscopic agglutination test (MAT) panel at the WHO/FAO/OIE Collaborating Centre for Reference and Research on Leptospirosis, Brisbane, Australia.**
Additional file 2:
**Locally acquired leptospiral serovars isolated from humans, Queensland 2000 to 2013.**

## Abbreviations

CAAT: Cross-agglutination absorption test
CI: Confidence interval
GIS: Geographic information systems
MAT: Microscopic agglutination test
PCR: Polymerase chain reaction
WHO/FAO/OIE: World Health Organization/Food and Agriculture Organization/World Organisation for Animal Health
