# Supplementary material for: The emergence of Leptospira borgpetersenii serovar Arborea in Queensland, Australia, 2001 to 2013
Source: BMC Infect Dis. 2015 Jun 14;15:230. doi: 10.1186/s12879-015-0982-0 (PMC4465322; doi:10.1186/s12879-015-0982-0)
Supplement: Additional file 1: — Leptospiral serovars used in the routine microscopic agglutination test (MAT) panel at the WHO/FAO/OIE Collaborating Centre for Reference and Research on Leptospirosis, Brisbane, Australia. [file 12879_2015_982_MOESM1_ESM.doc]

**Additional file 1**

Leptospiral serovars used in the current routine microscopic agglutination test (MAT) panel at the WHO/FAO/OIE Collaborating Centre for Reference and Research on Leptospirosis, Brisbane, Australia.

| **Species** | **Serovar** | **Strain** |
| --- | --- | --- |
| *L. interrogans* | Pomona | Pomona |
| *L. interrogans* | Hardjo | Hardjoprajitno |
| *L. borgpetersenii* | Tarassovi | Perepelitsin |
| *L. kirschneri* | Grippotyphosa | Moskva V |
| *L. weilii* | Celledoni | Celledoni |
| *L. interrogans* | Copenhageni | M20 |
| *L. interrogans* | Australis | Ballico |
| *L. interrogans* | Zanoni | Zanoni |
| *L. interrogans* | Robinsoni | Robinson |
| *L. interrogans* | Canicola | Hond Utrecht IV |
| *L. interrogans* | Kremastos | Kremastos |
| *L. interrogans* | Szwajizak | Szwajizak |
| *L. interrogans* | Medanensis | Hond HC |
| *L. kirschneri* | Bulgarica | Nicolaevo |
| *L. kirschneri* | Cynopteri | 3522C |
| *L. borgpetersenii* | Arborea | Arborea |
| *L. interrogans* | Bataviae | Swart |
| *L. interrogans* | Djasiman | Djasiman |
| *L. borgpetersenii* | Javanica | Veldrat Batavia 46 |
| *L. noguchii* | Panama | CZ 214 |
| *L. santarosai* | Shermani | 1342K |
| *L. weilii* | Topaz | 94-79970/3 |
