# Supplementary material for: The emergence of Leptospira borgpetersenii serovar Arborea in Queensland, Australia, 2001 to 2013
Source: BMC Infect Dis. 2015 Jun 14;15:230. doi: 10.1186/s12879-015-0982-0 (PMC4465322; doi:10.1186/s12879-015-0982-0)
Supplement: Additional file 2: — Locally acquired leptospiral serovars isolated from humans, Queensland 2000 to 2013. [file 12879_2015_982_MOESM2_ESM.doc]

**Additional file 2**

Locally acquired leptospiral serovars isolated from humans, Queensland 2001 to 2013.

| **Species** | **Serovar** |
| --- | --- |
| *L. borgpetersenii* | Arborea |
| *L. interrogans* | Australis |
| *L. weilii* | Celledoni |
| *L. borgpetersenii* | Ballum |
| *L. interrogans* | Bindjei |
| *L. interrogans* | Broomi |
| *L. weilii* | Celledoni |
| *L. interrogans* | Hardjo |
| *L. interrogans* | Kremastos |
| *L. interrogans* | Mankarso |
| *L. interrogans* | Robinsoni |
| *L. interrogans* | Szwajizak |
| *L. weilii* | Topaz |
| *L. interrogans* | Zanoni |
